# Pathway of Green Development of Yangtze River Economics Belt from the Perspective of Green Technological Innovation and Environmental Regulation

**DOI:** 10.3390/ijerph181910471

**Published:** 2021-10-05

**Authors:** Tifang Ye, Hao Zheng, Xiangyu Ge, Keling Yang

**Affiliations:** 1 Institute of Information Management and Statistics, Hubei University of Economics, Wuhan 430205, China; yetifang@hbue.edu.cn (T.Y.); ykl19810104@hbue.edu.cn (K.Y.); 2 School of Statistics and Mathematics, Zhongnan University of Economics and Law, Wuhan 430073, China; zhenghao@stu.zuel.edu.cn

**Keywords:** Yangtze River Economic Belt, green technological innovation, eco-efficiency, environmental regulation, super-efficiency SBM window model

## Abstract

The eco-efficiency of the Yangtze River Economic Belt from 2005 to 2019 has been evaluated by the super-efficiency SBM window model, the results of which are taken as the measurement standard for green development. Next, more attempts have been done to figure out the impacts of green technological innovation on the green development in urban clusters of the Yangtze River Economic Belt by a systematic GMM model, further confirming the moderation effect of dual environmental regulations on the relationship between green technological innovation and green development and the heterogeneity in different urban clusters of the Yangtze River Economic Belt. Finally, it is a fact that a cascade has been pointed out in green development of the Yangtze River Economic Zone. With an empirical analysis, it has been found that green technological innovation has a positive moderating effect on green development in the downstream regions, and the relationship between green technological innovation and green development is positively affected by the formal environmental regulations in the overall Yangtze River Economic Zone and the midstream region. Meanwhile, informal environmental regulations play a positive role in moderating the links between green technological innovation and green development in the overall Yangtze River Economic Zone, as well as the upstream, midstream and downstream regions. Based on the conclusions of the research, some policy suggestions of a multi-environmental regulation governance system and regional differentiated environmental regulation are given at last.

## 1. Introduction

Green development is expected to become a new driving force in the 14th Five-Year Plan period to promote sustainable and high-quality economic development. As the support belt of China’s economy, named for the complete green ecological corridor of the Yangtze River Basin, the Yangtze River Economic Belt spans the three regions of East and West China, covering nine provinces and two cities. As one of the “three strategic” regions implemented by the central government, as well as an inland economic belt with global influence, famous for a coordinated development belt with interaction and cooperation between east and west, the Yangtze River Economic Belt is also a comprehensive development belt for opening up to the outside world along the coast and rivers, as well as an early demonstration belt for ecological civilization construction, with a high economic and ecological status. Therefore, it is of great significance to explore the green development of the Yangtze River Economic Belt.

Recently, although the Chinese government has issued a series of policies and regulations on “promoting green development and building a market-oriented green technological innovation system” to stimulate green economy and green technological innovation as the cornerstone of green development, it is clear to see the inadequacy in green technological innovation. Previous studies have shown that appropriate environmental regulations are proven to stimulate technological innovation and improve competitive advantage [1]. Environmental regulations have offered a breakthrough to promote green technological innovation. However, the environmental regulations have limited the impact to regional green technological innovation, which is as limited as the regional green technological innovation to green development. With a full summation of the previous relevant literatures on the relationship between environmental regulation, green technological innovation and green development, it can be divided into two aspects as follows.

Firstly, the focus has been put on the relationship between environmental regulation and green technological innovation. It has been found that the impact of environmental regulation on green technological innovation has two sides. (i) Environmental regulation operates in favor of green technological innovation, the results of which are mostly based on the Porter hypothesis, suggesting that environmental regulation can promote green technological innovation and the diffusion of enterprises through the “innovation compensation” effect [2,3,4]. The realization of external pressure exerted by environmental regulation can effectively overcome organizational inertia and complement the internal governance mechanisms of firms, transforming external pressure into an incentive factor to promote technological innovation activities, which were presented by Ambec and Barla [5]. (ii) Environmental regulation has the tendency to inhibit green technological innovation. One study by Clarkson et al. [6] found that environmental regulation has increased the cost of pollution control and the institutional compliance of firms. An analysis of the firm involved in production cuts and shutdowns under severe environmental regulation to reduce the funds available for innovation was carried out by Petroni et al. [7]. (iii) The impact of environmental regulation on green technological innovation is uncertain. Due to the different intensities of environmental regulations, different development levels of industry and heterogeneity of environmental regulation tools, the impact of environmental regulations on green technological innovation is uncertain. The research by Li et al. [8] found that the impact of environmental regulation on green technological innovation showed a “U” shape as its intensity increased. Yuan et al. [9] studied the impact of environmental regulation on green technological innovation in manufacturing and found that, with different levels of eco-efficiency, the impact of environmental regulation on green technological innovation also showed different effects. The founding by Feng and Chen [10] discovered that different types of environmental regulation tools have different impacts on industrial green technology innovation. Yu and Cui [11] estimated that the effect of formal environmental regulation on technological innovation presents an inverted “N” relationship, while the effect of informal environmental regulation on technological innovation weakens as its intensity increases. A study from a micro-firm perspective by Li and Xiao [12] offered a comprehensive analysis that heterogeneous environmental regulation tools significantly differed in their incentives for green innovation.

Secondly, the emphasis of exploring research of the relationship between green technological innovation and green development was proposed. Meanwhile, as more attention has been paid to green development, more and more studies focus on the impact of green technological innovation on economy and society at different levels. (i) From the macro level, research has mainly focused on the relationship between green technological innovation and regional green development. Green technological innovation is beneficial for increasing green consumption to promote the development of regional green economy [13,14]. (ii) From the meso level, research has mainly focused on the relationship between green technological innovation and industrial sustainable development. A study by Yuan and Zhang [15] found that green technological innovation (technological caused by environmental regulations) is significantly and positively related to innovation industrial sustainable development. A preliminary work on a U-shaped relationship between green technological innovation and China’s manufacturing transformation and upgrading was undertaken by Yuan and Chen [16], which appeared as regional differences in the east, middle and west of China. (iii) From the micro level, research has mainly focused on the relationship between corporate green technological innovation and sustainable development performance. Research by Chang [17] concluded that the “isolation mechanism” of green technological innovation can protect the company’s marginal profit and obtain revenue. A study discovered by Fernando et al. [18] showed that green technological innovation can significantly affect the company’s sustainable organizational performance.

Thirdly, it can be seen that few literature cover the relationship between green technological innovation to green development under the current stage of China, and research on the Yangtze River Economic Belt, an important strategic place in China, is almost a blank. On the other hand, there is no general agreement on the economic effects of environmental regulation in academia, lacking research on the moderating effect of environmental regulation on the relationship between green technological innovation and green development. Based on the background of the prevalence of green development and ecological civilization, it aims to investigate whether green technological innovation in the Yangtze River Economic Zone effectively supports green development and examine whether heterogeneous environmental regulations in the Yangtze River Economic Zone can stimulate enterprises’ willingness to innovate green technology to confirm whether they play a moderating role in the relationship between green technological innovation and green development. Is there heterogeneity of the moderating effect in the upper, middle and lower reaches of the Yangtze River Economic Belt? The contributions of this paper are mainly shown in the following aspects.

First, the super-efficiency SBM window model is applied to evaluate the comparable eco-efficiency of the Yangtze River Economic Belt on the cross-section and time series, taking it as a measure of the regional green development to analyze the green development of different urban areas of the Yangtze River Economic Belt from 2005 to 2019 through spatial and temporal dynamics, which have expanded and enriched the related research.

Second, a system generalized moment model is constructed empirically. With the two-step system generalized moment estimation method (two-step SYS-GMM), it is designed to test whether the regional green technological innovation can effectively promote regional green development even in a spatial heterogeneity analysis.

Third, an empirical analysis is undertaken to test the performance of heterogeneous environmental regulations in moderating the relationship between green technological innovation and green development with respect to formal and informal environmental regulations, respectively, to clarify the role of the construction of a multi-environmental regulation governance system in promoting green technological innovation and green economic development on a regional level.

The structure of this paper is organized as follows: the theoretical mechanism analysis is located in Section 2. In Section 3, we discuss the research design of this paper, including the selection and measurements of the research indicators and the construction of the theoretical model. The empirical results of this paper are analyzed in Section 4, followed by various robustness tests in Section 5. Finally, the last section is the conclusions and policy recommendations of this paper.

## 2. Theoretical and Mechanistic Analysis

So-called green development not only emphasizes the increase of green economic output but also the reduction of pollution emissions. Environmental regulation is a social regulation aimed at reducing pollution emission to protect the environment. From the different perspectives of implementors, environmental regulations can be divided into formal environmental regulations and informal environmental regulations [11]. The administration of formal environmental regulations is the government, issuing commands and controls with executive orders, laws and regulations, with the type of economic incentive and constraint with means of market regulation [19] specifically including, but not limited to, the collection of sewage charges, punishment of environmental administrative, financial expenditures for environmental protection and promulgation of environmental laws and regulations. Different environmental regulation tools are effective to remedy the polluting behavior of enterprises either through economic means such as fiscal taxes and fees or administrative means like commands and controls [20]. The concept of informal environmental regulation was firstly proposed by Pargal and Wheeler [21], referring to entering into the negotiation or consultation of pollution reduction between the public, media or social groups and enterprises with the behavior of polluting with a view toward environmental protection, including complaints and accusations from residents, the resistance toward enterprises with acts of polluting, social opinion pressure and a joint boycott of products from these enterprises, which is generally related to the public’s environmental protection concept, environmental protection consciousness, environmental protection attitude and awareness. It has a binding force toward irregular behaviors within or beyond the scope of formal regulation, which is an important power to supervise the operation of enterprises [11] and an effective supplement for formal environmental regulation.

In this subsection, firstly, from the micro perspective, representative firms and consumers are designed to analyze the mechanism of environmental regulation in regulating the relationship between green technological innovation and green development by constructing a consumer utility function. On the basis of the above analysis, relevant hypotheses are proposed.

It is assumed that there are only two major categories of products in the market: green products g and the other products $x_{i}\left(i=1,2,....,I\right)$, whose prices are $p_{g}$ and $p_{i}$, respectively. The supply of firms A is only limited to green products g, with a range of firms $B_{i}\left(i=1,2,....,I\right)$ with products $x_{i}$, where I denotes all types of other products. At a given income level, consumers strive to plan two types of products to maximize their utilities, which can be expressed as follows.

(1) $$\max\;U\left(g,x_{i}\right)={\left[a{\left(p_{g}g\right)}^{\alpha }+b\int_{0}^{I}{\left(p_{i}x_{i}\right)}^{\alpha }di\right]}^{\frac{1}{\theta }}$$

(2) $$s.t\;Y=p_{g}g+\int_{0}^{I}\left(p_{i}x_{i}\right)di$$

where the utility function in Equation (1) is constructed on the basis of the utility function proposed by Dixit and Stiglitz for diversified products [22], and α,θ respectively representing the parameters of the elasticity of substitution for green and other products. Assuming that there is no possibility of constant and increasing returns to scale of green products and consumption, it satisfies 0<α<θ<1. The level of consumer spending in Equation (2) is expressed by the per capita budget constraint. Then, a,b>0 respectively correspond to the level of utility resulting from the purchase of green and non-green products by consumers.

According to the principle of utility maximization, it is necessary to respectively differentiate the green product g and other goods $x_{i}\left(i=1,2,....,I\right)$ in Equation (1) to obtain the expression of the other goods $x_{i}={\left(\frac{a}{b}\right)}^{\frac{1}{\alpha -1}}\frac{p_{g}g}{p_{i}}$, substituting them into Equation (2) to calculate the demand of green products and other products as follows:

(3) $$g=\left(\frac{Y}{p_{g}}\right)/\left(1+\frac{I}{\lambda }\right),\;x_{i}=\left(\frac{Y}{p_{i}}\frac{1}{\lambda }\right)/\left(1+\frac{I}{\lambda }\right)$$

where $\lambda ={\left(\frac{a}{b}\right)}^{\frac{1}{1-\alpha }}$.

What matters is the regulatory mechanism of the dual environmental regulation on the relationship between green technological innovation and green development. Two scenarios are clearly considered as the following.

Scenario 1: In the early stage of green technological innovation, due to the high investment and risk, enterprises of green technological innovation are obviously going to fail to have a price advantage, so $p_{g}>p_{i}$. According to Equation (3), it is possible to $g<x_{i}$, which means a smaller demand for green products than the other non-green products. For this reason, the fewer a green industry enterprise is, the lower a level of green technology production is, and so is the green economic output. Rarely does the production department use clean energy technology, along with worse pollution emission. Therefore, with the lower level of green development, it is clear to know the green technological innovation without making a significant contribution to green development.

Regarding formal environmental regulation, we consider the following two cases in Scenario 1: In the first case, considering that the regional government adopts a formal environmental regulatory policy that focuses on subsidies such as environmental protection fiscal expenditures, which encourages green enterprises to lower the price of green products, then $p_{g}\leq p_{i}$. According to Equation (3), it occurs as $g\geq x_{i}$. In other words, the demand for green products is greater than or equal to that for non-green products, which sends a signal to enterprises to stress green technological innovation. Therefore, more and more enterprises join the team of green production, coming to the conclusion of increasing the green economic output and improving green development, which explains that formal environmental regulation positively moderates the relationship between green technological innovation and green development. In the second case, if the regional government adopts a formal environmental regulatory policy that focuses on sewage charges, environmental administrative penalties or environmental laws with unsuitable intensity for the development of the local economy and enterprises, the enterprise is preferred to keep to the “compliance cost” effect to lower profits. To survive, green enterprises choose the way of raising the prices of green products; then, $p_{g}>p_{i}$. Therefore, the formal environmental regulation is not successful at moderating the relationship between green technological innovation and green development in this case.

Furthermore, regarding informal environmental regulation, there are also two cases in Scenario 1: In the first case, when the public’s environmental awareness has not been awakened and the informal environmental regulation is at a low level, leading to not particularly favorable green products with no price advantage, the utility brought by consuming green products tends to be less than that by non-green products; then, a<b. According to the expression of λ, there must be λ<1, namely, 1/λ>1. It comes to $g<x_{i}$ referring to Equation (3). It is a fact that the demand for green products must be less than the demand for non-green products. Therefore, the informal environmental regulation is not successful at moderating the relationship between green technological innovation and green development in this case. In the second case, when the informal environmental regulation reaches a certain level, meanwhile, the public’s environmental awareness is relevant to a certain extent; the utility brought by consuming green products tends to be greater than non-green products brought; then, a>b and 0<1/λ<1. It comes to $g\geq x_{i}$ referring to Equation (3). It can be found that the demand for green products must be greater than the demand for non-green products, in line with the results of the first case regarding formal environmental regulation, which reflects informal environmental regulation and positively moderates the relationship between green technological innovation and green development in this case.

Scenario 2: When green technological innovation has been progressed at a new stage, the enterprises of green technological innovation has passed through the startup phase. The gradual cost reduction of green production can be realized by mature skills of the green production, then the price $p_{g}$ goes down dramatically, and $p_{g}<p_{i}$. According to Equation (3), it is more likely to get $g>x_{i}$, stating that the demand of green products is greater than that of the other non-green products. At this time, the higher the level of green technology production is, the better the output of green economy is. However, it is not supposed to improve the performance of pollution emission in fewer green production sectors, and green technological innovation seems not to boost the green development. However, the more enough green industry enterprises are, the higher the level of green technology production will be, and so will the output of green economy. With the increasing number of sectors with clean technology production, the pollution emission will be improved more to promote the level of green development. In consequence, green technological innovation will have a significant promotion effect on green development.

Similar to the analysis in Scenario 1, we consider the following two cases regarding formal environmental regulation: In the first case, considering that the regional government adopts a formal environmental regulatory policy that focuses on subsidies such as environmental protection fiscal expenditures, it comes to the drop of $p_{g}$, and it is a greater likelihood of $g\geq x_{i}$, which is similar to the details of the first case in Scenario 1, which is the formal environmental regulation positively moderates the relationship between green technological innovation and green development in this case. In the second case, considering that the regional government adopts a formal environmental regulatory policy that focuses on sewage charges, environmental administrative penalties or environmental laws with unsuitable intensity toward the development of the local economy and enterprises. In order to make profits, green enterprises will definitely increase the price of green products, ending with a higher probability of $p_{g}\leq p_{i}$ and $g<x_{i}$, which are similar to the details of the second case in Scenario 1, which is that the formal environmental regulation is not successful at moderating the relationship between green technological innovation and green development in this case.

Similar to the analysis in Scenario 1, we consider the following two cases regarding informal environmental regulation: In the first case, the informal environmental regulation is at a low level; then, a<b. According to the expression of λ, it must be λ<1; then, it becomes $g<x_{i}$ referring to Equation (3). It is obvious that the demand for green products must be less than the demand for non-green products. As a result, the informal environmental regulation is not successful at moderating the relationship between green technological innovation and green development in this case. In the second case, when the informal environmental regulation reaches a certain level, a>b and 0<1/λ<1. It becomes $g\geq x_{i}$, referring to Equation (3). The fact is that the demand for green products must be greater than the demand for non-green products, so that informal environmental regulation positively moderates the relationship between green technological innovation and green development in this case.

Based on the above analyses in Scenario 1 and Scenario 2, two hypotheses are proposed as follows:

**Hyphotesis** **1** **(H1).** *When green technological innovation is at a low level, it will have no significant effect on green development. When green technological innovation is at a high level, a significant positive effect will be exerted on green development*.

**Hyphotesis** **2** **(H2).** *Formal environmental regulation may positively regulate the relationship between green technological innovation and green development, which mainly depends on whether the type and intensity of formal environmental regulation are suitable for the development of local economy and enterprises, as does informal environmental regulation, with dependence on the intensity of informal environmental regulation*.

**Figure 1:**
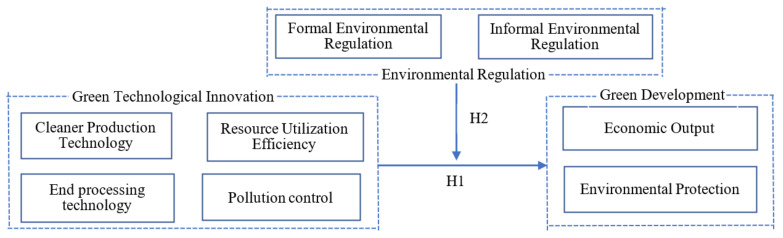
Diagram of the theoretical and mechanistic analyses.

In summary, the theoretical and mechanistic analyses of the hypotheses constructed in this paper are shown in Figure 1.

## 3. Study Design

Debt risks have become a significant challenge in the economic growth, which stems from the contradiction between the uncertainty of asset income and the sustainability of debt repayment. Comprehensively, the local government debt risks are not only closely linked to the liabilities of local government but also the assets of the local government. In addition, the assets used for debt repayment are considered as the effective guarantee of the local governments’ solvency. Therefore, it is necessary to compile a macro-balance sheet of local governments to measure the local government debt risks of various provinces in China in an all-round way.

### 3.1. Measurement of Green Development

According to the available literature, three main methods are mentioned to measure green development. The first method is the single indicator method. For instance, the per unit of output of natural resource consumption reported by Cao and Ren [23], per unit of output of environmental pollution proposed by Liu and Wen [24] and per unit of GDP of energy consumption applied by Wen et al. [25]. The second method is the index system method. For instance, the “Wealth Accounting in Ecosystem Service Evaluation” proposed by the World Bank [26] includes a green economy indicator system, the green economy indicator system proposed by the EEA (see European Environment Agency [27]). The third method is the efficiency method, which actually has been operated with the SBM model of undesirable outputs, with selecting input and output indicators in the following core features of green development, the aim of which is to achieve higher economic output with fewer resource inputs while as little environmental pollution as possible. Of course, it is related to the concepts of eco-efficiency [28,29,30], environmental efficiency [31,32] and green economic efficiency [33,34]. The SBM model of undesirable output is more widely used, because it is weighed objectively with a combination of multiple input information to take both the desirable output and undesirable output into consideration and the realization of the desirable output represented by GDP and undesirable output represented by environmental pollution. Furthermore, SBM is a non-radial model that allows for nonproportional reductions in each input or augmentations in each output. In other words, it is accurate to make an efficiency score for each input and each output, which is the reason why SBM has been proposed instead of directional distance functions (DDFs). In addition, some specific discussions have been made to explain why do not choose stochastic frontier analysis (SFA) is superior to measure eco-efficiency. As we all know, SFA, a parametric method, is necessary to set the form of production function in advance so that the result may not be consistent with the actual situation. Moreover, the distribution form of the inefficiency term is required to be determined without handling the multi-output situation. As a nonparametric method, the form of the frontier production function is not needed by SBM, which can effectively deal with the situation of multiple inputs and multiple outputs. In addition, the effectiveness has nothing to do with the units of the variables. In conclusion, taking the core characteristics of green development in account, as the regional ecological efficiency is designed as a measurement of regional green development, SBM is deemed to be the basic model for measuring eco-efficiency.

#### 3.1.1. Super-Efficiency SBM Model

As for the assessment of eco-efficiency, the Slacks-Based Measures (SBM) Model proposed by Tone [35] aims at measuring the eco-efficiency for undesirable outputs such as environmental pollution. In most models of data envelopment analysis (DEA), the best performer refers to the one with full efficient status denoted by unity (or 100%). Generally speaking, plural decision-making units (DMUs) are the best manifestation of the “efficient status”. Later, the super-efficiency SBM was proposed by Tone (2002) [36], which appears to discriminate these efficient DMUs. The super-efficiency SBM integrates the advantages of the super-efficient DEA model and the SBM model. It clearly makes further discriminations between the efficient DMUs at the frontier under the conditions of the undesirable outputs. In this paper, based on the super-efficiency SBM model by Tone [36], each region is regarded as a DMU with a total of *n*. DMUs, and each DMU is equipped with *m* input (including the capital input, physical resource input and human resource input); a desired output $s_{1}$ and an undesirable output $s_{2}$, which are represented respectively by the matrices as $X=\;\left(x_{ij}\right)\in R^{m\times n}$, $Y^{g}=\left(y_{ij}^{g}\right)\in R^{s_{1}\times n}$ and $Y^{b}=\left(y_{ij}^{b}\right)\in R^{s_{1}\times n}$, where $x_{ij}$ is expressed by the i input j DMU, $y_{ij}^{g}$ by the *i* desired output the j DMU and $y_{ij}^{b}$ by the *i* undesirable output j DMU. The super-efficiency issue has been discussed under the assumption that the DMU $\left(x_{0},y_{0}\right)$ is SBM-efficient. Let a production possibility set $P\backslash \left(x_{0},y_{0}\right)$ as 


$$P\backslash \left(x_{0},y_{0}\right)=\left\{\left(\bar{x},\overset{–}{y}\right)|\bar{x}\geq \sum_{j=1,\neq 0}^{n}\lambda_{j}x_{j},\overset{–}{y}\leq \sum_{j=1,\neq 0}^{n}\lambda_{j}y_{j},\overset{–}{y}\geq 0,\lambda \geq 0\right\}$$

Further, a subset $\bar{P}\backslash \left(x_{0},y_{0}\right)$ of $P\backslash \left(x_{0},y_{0}\right)$ is defined as

$$\bar{P}\backslash \left(x_{0},y_{0}\right)=P\backslash \left(x_{0},y_{0}\right)\cap \left\{\bar{x}\geq x_{0}\mathrm{and}\overset{–}{y}\leq y_{0}\right\}$$

The super-efficiency of $\left(x_{0},y_{0}\right)$ is defined as the optimal objective function value $\delta^{*}$ of the following Model (4):

(4) $$\delta^{*}=\min\frac{\frac{1}{m}\sum_{i=1}^{m}\frac{{\bar{x}}_{i}}{x_{i0}}}{\frac{1}{s_{1}+s_{2}}\left(\sum_{r=1}^{s_{1}}\frac{{\bar{y}}_{r}^{g}}{y_{r0}^{g}}+\sum_{r=1}^{s_{2}}\frac{{\bar{y}}_{r}^{b}}{y_{r0}^{b}}\right)}\;s.t.\left\{\begin{array}{c} \bar{x}\geq \sum_{j=1,\neq 0}^{n}\lambda_{j}x_{j}\\ {\bar{y}}^{g}\leq \sum_{j=1,\neq 0}^{n}\lambda_{j}y_{j}^{g}\\ {\bar{y}}^{b}\geq \sum_{j=1,\neq 0}^{n}\lambda_{j}y_{j}^{b}\\ \bar{x}\geq x_{0},{\bar{y}}^{g}\leq y_{0}^{g},{\bar{y}}^{b}\leq y_{0}^{b},\lambda \geq 0 \end{array}\right.$$

where λ refers to the weight vector, and $\left(x_{0},y_{0}^{g},y_{0}^{b}\right)$ are denoted as the input, desired output and undesirable output of a certain DMU.

According to the analysis by Tone [36], let $\left(\alpha x_{0},\beta y_{0}\right)$ with α≤1 and β≥1 be a DMU with reducing inputs, followed by enlarging the desirable outputs and reducing the undesirable outputs as $\left(x_{0},y_{0}\right)$. Then, the super-efficiency score of $\left(\alpha x_{0},\beta y_{0}\right)$ is not less than that of $\left(x_{0},y_{0}\right)$. The fractional program, i.e., super-efficiency SBM can be transformed into a linear programming problem by Charnes–Cooper transformation as [37,38]:

(5) $$\tau^{*}=\min\tau =\frac{1}{m}\sum_{i=1}^{m}\frac{{\bar{x}}_{i}}{x_{i0}}\;s.t.\left\{\begin{array}{c} 1=\frac{1}{s_{1}+s_{2}}\left(\sum_{r=1}^{s_{1}}\frac{{\tilde{y}}_{r}^{g}}{y_{r0}^{g}}+\sum_{r=1}^{s_{2}}\frac{{\tilde{y}}_{r}^{b}}{y_{r0}^{b}}\right)\\ \tilde{x}\geq \sum_{j=1,\neq 0}^{n}\Lambda_{j}x_{j}\\ {\tilde{y}}^{g}\leq \sum_{j=1,\neq 0}^{n}\Lambda_{j}y_{j}^{g}\\ {\tilde{y}}^{b}\geq \sum_{j=1,\neq 0}^{n}\Lambda_{j}y_{j}^{b}\\ \tilde{x}\geq tx_{0}\mathrm{and}\tilde{y}\geq ty_{0}\\ \Lambda \geq 0,\tilde{y}\geq 0,t>0 \end{array}\right.$$

According to the analysis by Tone [36], let an optimal solution of Model (5) be $\left(\tau^{*},{\tilde{x}}^{*},{\tilde{y}}^{*},\Lambda^{*},t^{*}\right)$. An optimal solution of the super-efficiency SBM can be expressed as

$$\delta^{*}=\tau^{*},\lambda^{*}=\Lambda^{*}/t^{*},{\bar{x}}^{*}={\tilde{x}}^{*}/t^{*},{\bar{y}}^{*}={\tilde{y}}^{*}/t^{*}$$

#### 3.1.2. Super-Efficiency SBM Window Model

Model (4) is useful to solve the problem of input and output slacks and discriminate efficient DMUs. However, Model (4) is a static model without performing cross-sectional efficiency of the DMUs over time, which can be addressed effectively by the DEA window analysis method.

The DEA window analysis method proposed by Charnes et al. [39] operates efficiency measurements by treating DMUs of a different period as different DMUs on the principle of the moving average. In the DEA window analysis framework, it compares the eco-efficiency of a DMU in a certain period not only with that of the other DMUs in the same period but also with its own eco-efficiency in other periods. The key thing to notice is the selection of window width *d*. According to the analysis of Charnes et al. [39], when the window width is sized to either *d* = 3 or *d* = 4, the result demonstrates a better balance in terms of reliability and stability of the efficiency measure. If there are DMUs of size *n* with the window width of *d*, it is certain to have DMU of size *d × n* in one window. On the assumption of the total time length of T, windows of size T − *d* + 1 for each DMU are required to measure the efficiency, with an efficiency value of size *d* in any window.

The moving average method shows that, for each DMU window, the efficiency values of size *d* on the first window are measured from the first time point *t* = 1. Then, moving to the second time point *t* = 2, the calculation of the efficiency values of size *d* on the second window are made from that time point. Finally, the efficiency values of size *d* on the last window are completed until the time point T − *d* + 1, but the final result of the evaluated DMU is predicted by the average efficiency value at each time point. The specific operation method is shown in Table 1.

A more closely relevant study by Wang and Feng [40], who constructed a DEA window analysis model to evaluate the inter-provincial energy efficiency in China, a super-efficiency SBM window model has been constructed in this paper. The eco-efficiency $E_{\mathrm{st}}$ of a DMU at the time *t*$\left(t=1,2,\cdots ,d\right)$ within the window *s* $\left(s=1,2,\cdots ,T-d+1\right)$ expressed by a super-efficiency SBM model is shown as follows:

(6) $$E_{\mathrm{st}}=\min\frac{\frac{1}{m}\sum_{i=1}^{m}\frac{{\bar{x}}_{i}^{st}}{x_{i0}^{st}}}{\frac{1}{s_{1}+s_{2}}\left(\sum_{r=1}^{s_{1}}\frac{{\bar{y}}_{r}^{g,st}}{y_{r0}^{g,st}}+\sum_{r=1}^{s_{2}}\frac{{\bar{y}}_{r}^{b,st}}{y_{r0}^{b,st}}\right)}\;s.t.\left\{\begin{array}{c} {\bar{x}}^{st}\geq \sum_{j=1,\neq 0}^{d\times n}\lambda_{j}^{st}x_{j}^{st}\\ {\bar{y}}^{g,st}\leq \sum_{j=1,\neq 0}^{d\times n}\lambda_{j}^{st}y_{j}^{g,st}\\ {\bar{y}}^{b,st}\geq \sum_{j=1,\neq 0}^{d\times n}\lambda_{j}^{st}y_{j}^{b,st}\\ {\bar{x}}^{st}\geq x_{0}^{st},{\bar{y}}^{g,st}\leq y_{0}^{g,st},{\bar{y}}^{b,st}\leq y_{0}^{b,st},\lambda_{j}^{st}\geq 0 \end{array}\right.$$

where j=1,2,⋯,d×n;s=1,2,⋯,T−d+1;t=1,2,⋯,d, the meanings of all the variables are basically in accordance with Model (4). More precisely, the superscript (subscript) st demonstrates the variable at the time point of *t* within the window of *s*. As an example of $x_{j}^{st}$, it indicates the input vector of the DMU *j* at the time of *t* within the window of *s*. In this paper, Model (6) is put in the evaluation of the comparable eco-efficiency of the Yangtze River Economic Zone urban agglomeration from 2005 to 2019 in both the cross-sectional and time series.

Regarding the characteristics of the ecological environment of the Yangtze River Economic Zone and the availability of relevant data, an index system is constructed to evaluate the ecological efficiency of the Yangtze River Economic Zone in the three dimensions of input, desired output and undesirable output shown in Table 2.

The indicators of the eco-efficient point to resource constraints mainly include the capital input and resource input. Among them, the capital input is converted into stock data based on 2005 by using the perpetual inventory calculation method. The resource input is mainly performed with involvement of energy, water resources, land resources and human resources. It prefers the option of energy consumption to the total amount of standard coal converted by electricity, gas and liquefied petroleum gas. The description of the built-up area is somehow provided by the intensive use of land in the region, which is accurate for measuring the consumption of land resources in this paper. In addition, the consumption of water resources is presented by the total amount of water consumption, while the human resource input is measured by employment.

The eco-efficiency output indicator is composed of desired output indicators and undesirable output indicators. The former is the regional development indicator measured by the gross regional product (GDP is converted to constant prices based on 2005 in the actual calculation), the latter for undesirable output indicators with environmental benefits. With a view toward the availability of the data, the environmental constraints are represented by industrial wastewater discharged, sulfur dioxide emission and industrial dust emission.

### 3.2. Variables and Measurement Models

#### 3.2.1. Core Explanatory Variables

Firstly, in accord with Valeria et al. [41], the measurement of the core explanatory variable of green technological innovation is based on the green patent IPC classification number in the “International Patent Green Classification List” launched by the World Intellectual Property Organization (WIPO) in 2010, which was made by the United Nations Framework Convention on Climate Change and is available on the website of WIPO at (https://www.wipo.int/classifications/ipc/en/green_inventory/ accessed on 15 June 2021). Green technology patents in China are shown as follows: biofuels, other thermal manufacturing or uses, rail vehicles, energy supply lines, general building insulation, recycling mechanical energy and wind and fuel cells. The search spreads out to cover applications and grants of green patents for each prefecture-level city every year in the Yangtze River Economic Zone at the website of the State Intellectual Property Office (SIPO) of China. Necessarily, the number of applications processing a green patent has been added to by one, and a natural logarithm is used to describe the green technological innovation level of each prefecture-level city. The greater the value is, the higher the level of green technological innovation in the prefecture-level city will be.

As for environmental regulation, it consists of formal environmental regulation and informal environmental regulation. The first method for measuring formal environmental regulation (reg) is the single-indicator method with a single indicator such as the amount of investment in environmental pollution control or the amount of sewage charges levied [16]. Another is the comprehensive index method synthesized by the entropy method to assign weights to indicators such as regional pollution emissions, the wastewater emission rate, sulfur dioxide removal rate, smoke and dust removal rate and solid waste utilization rate [42,43]. Based on the theoretical and mechanistic analysis in the second part of this paper, the second approach is more suitable and subjective by the entropy method, which is an objective method to determine the weights of the indicators according to the amount of the information provided by the observations of each indicator. The greater the amount of information, the smaller the uncertainty will be. In other words, it shows smaller entropy, followed by a greater degree of dispersion, so it comes with a greater weight of the indicator. To assign weights to the removal rate of industrial dust, we used the comprehensive utilization rate of industrial solid waste, the removal rate of sulfur dioxide and the harmless treatment rate of household garbage. The methods for measuring informal environmental regulation (ireg) are discussed in the following: the first method was attributable to the widespread of internet search data. For example, Google public concern and Baidu news coverage reported by Xu [44] and the number of news comments on haze pollution from Baidu used by Zhang et al. [45]. The second method was to construct the public demand index with information of the number of environmental letters and visits, proposals and suggestions from NPC deputies and CPPCC members [46,47]. The third method, proposed by Pargal and Wheeler [21], was a method of comprehensive index synthesized by the entropy method to assign weights to indicators, such as per capita income, education level, population density and age structure, extensively used by Yu and Cui [11] and Féres and Reynaud [48]. On the ground of the theoretical and mechanistic analysis in the second part of this paper and the availability of relevant data in prefecture-level cities, the third method was chosen to measure informal environmental regulation with the application of the entropy method to integrate information on the per capita income and population density and the level of education, where the level of education was measured by the proportion of the sum of full-time teachers of higher education, ordinary middle schools and elementary schools in the total population at the end of the year.

#### 3.2.2. Control Variables

Even though the economic development and economic growth rate are associated with environmental quality [49] [50], the real GDP per capita (pgdp) and real GDP growth rate (r_gdp) in the model are useful to control the influence of economic growth factors on the eco-efficiency. According to the analysis of Jorgenson [51], less-developed countries appear to be “pollution refuges” to environmental degradation, with rapid economic development through the FDI. Then, the proportion of foreign investment in the model is needed to define the regional openness (open) to control the impact of openness on the eco-efficiency. With reference to Yu et al. [50], we added the proportion of the secondary industry (structure) to the model to capture the impact of the industrial structure on the eco-efficiency. In addition, according to the research of Ge et al. [52], urbanization has a significant impact on pollution emissions, and we added the urbanization rate (r_urb) to the model to capture the effect of urbanization on the eco-efficiency.

#### 3.2.3. Model Setting

Based on the analysis of the theoretical framework, unlike previous studies on the effectiveness of environmental regulation policies, the purpose of this paper focuses on the discussion on green technological innovation in each region to wonder if the intensity of the formal and informal environmental regulations is effective to stimulate the local green technological innovation to make progress in local green development. Therefore, attempts were made to construct a benchmark model of the impact of green technological innovation on green development to understand whether the green technological innovation in each region can effectively accelerate the local green development. In empirical practice, the formal and informal environmental regulations in each region enhance the willingness of green technological innovation to further promote local green development.

Based on the above ideas, eco-efficiency is defined as the explanatory variable, while green technological innovation is the core explanatory variable in the benchmark model. It is known that endogeneity is one of the important factors affecting the reliability of the model. According to the empirical model and the practical implications of this paper, it seems to figure out two main obstacles: the first is the missing variables. Although more than enough control variables affecting eco-efficiency were considered in the model, it is inevitable to leave out some variables related to the core explanatory variables of green technological innovation in the model, for example, green finance and research funding input due to the difficulty of data acquisition. The missing variables lead to a strong correlation between the residual terms and the explanatory variables as the problem of endogeneity. Secondly, it relates to the existence of bidirectional causality in the model, which means reason–result relationships between the explanatory variables and the explained variables. On one hand, it is an amazing accomplishment achieved with generous support from the regional government for green technological innovation with the use of alternative energy sources, process improvement and resource recycling to raise the local eco-efficiency. On the other hand, regions with higher eco-efficiency are generally the synthetic effect of superior natural endowments, higher levels of technological and economic development and higher cultural levels and literacy of residents, which influence the level of local green technological innovation. With full consideration of these issues, eco-efficiency takes the one period lag in the dynamic model estimation in the light of the system generalization matrix proposed by Arellano and Bover [53], while the first-order and second-order lagged terms of the endogenous variables are used as instrumental variables to eliminate the biased effects of endogeneity in the model. Thus, the model for dynamic panel regression is constructed as follow Model (7):

(7) $${\mathrm{EE}}_{it}=\alpha_{0}+\alpha_{1}{\mathrm{EE}}_{it-1}+\alpha_{2}\ln({\mathrm{tgreen}}_{it}+1)+X_{it}^{\prime }\alpha_{3}+\omega_{1i}+\mu_{1t}+\varepsilon_{1it}$$

where the subscripts i,t are denoted as prefecture-level cities and years and $\omega_{1},\mu_{1}$ correspond to the prefecture-level city and fixed effects of time, with $\varepsilon_{1}$ for error terms. Besides, EE is defined as eco-efficiency, which is used to measure the level of green development of the region. As we can see, tgreen signifies the number of green patent applications, but X is for the control variable. On the basis of the theoretical analysis, the impact of green technological innovation on green development can be changed over different environmental regulations. Therefore, the product terms of environmental regulation and green technological innovation are introduced to examine whether the impact of green technological innovation on green development is positively regulated by the environmental regulation policy in the Yangtze River Economic Belt. Therefore, the further model is constructed as follows:

(8) $${\mathrm{EE}}_{it}=\beta_{0}+\beta_{1}{\mathrm{EE}}_{it-1}+\beta_{2}\ln({\mathrm{tgreen}}_{it}+1)\times {\mathrm{reg}}_{it}+X_{it}^{\prime }\beta_{3}+\omega_{2i}+\mu_{2t}+\varepsilon_{2it}$$

(9) $${\mathrm{EE}}_{it}=\gamma_{0}+\gamma_{1}{\mathrm{EE}}_{it-1}+\gamma_{2}\ln({\mathrm{tgreen}}_{it}+1)\times {\mathrm{ireg}}_{it}+X_{it}^{\prime }\gamma_{3}+\omega_{3i}+\mu_{3t}+\varepsilon_{3it}$$

where reg in Model (8) represents formal environmental regulation, and ireg in Model (9) represents informal environmental regulation. Combining the estimation results of Model (7), Model (8) and Model (9), we can get the conclusions as follows: (i) If $\alpha_{2}$ and $\beta_{2}$ (or $\gamma_{2}$) are positive and significant, it can be derived that green technological innovation has a positive effect on the eco-efficiency, which will increase with the strengthening of the formal (or informal) environmental regulations. (ii) If $\alpha_{2}$ is not significant or $\alpha_{2}$ is negative and significant, along with $\beta_{2}$ (or $\gamma_{2}$) as positive and significant, it can be derived that the effect of green technological innovation on the eco-efficiency depends on the formal (or informal) environmental regulations. (iii) If $\beta_{2}$ (or $\gamma_{2}$) is not significant, the finding is that the formal (or informal) environmental regulations make no difference on the relationship between green technological innovation and the eco-efficiency. (iv) If $\beta_{2}$ (or $\gamma_{2}$) is negative and significant, it means that the formal (or informal) environmental regulations may weaken the effect of green technological innovation on the eco-efficiency.

#### 3.2.4. Sample Data

The selected indicators stem from the China Urban Statistical Yearbook from 2005 to 2019, in which the data of a fixed asset investment price index and GDP deflator are at the national level, and the green patent data is from the State Intellectual Property Office of China.

From the “Outline of the Yangtze River Economic Belt Development Plan” issued in September 2016, the Yangtze River Economic Belt covers in a grand total of 11 provinces. Owing to the classification standard of the “Guidance of the State Council on Promoting the Development of Yangtze River Economic Belt by Relying on the Golden Waterway” (2014), 31 cities located in the upstream area refer to the three provinces of Yunnan, Guizhou and Sichuan and one municipality of Chongqing. In view of the change in administrative division in 2011, Bijie and Tongren in Guizhou Province adjusted to Bijie City and Tongren City, so it excluded Bijie and Tongren. Due to the insufficient data of the three provincial cities of Tianmen, Xiantao and Qianjiang, the midstream region includes the 36 cities of Hubei, Hunan and Jiangxi, except those three cities. After completing the missing data of individual cities in individual years by interpolation, a total of 41 cities of Jiangsu, Zhejiang, Anhui and one municipality of Shanghai belong to the downstream region. Ultimately, balanced panel data of 1620 samples from 108 prefecture-level and above cities of the Yangtze River Economic Belt in 2005 and 2019 were realized.

## 4. Analysis of Empirical Results

### 4.1. Spatial and Temporal Variation of Regional Eco-Efficiency

#### 4.1.1. Time Dynamic Analysis

The Economic Belt has different maturities upstream, midstream and downstream, including the Yangtze River Delta Urban Agglomeration, Wuhan City Circle, Chang-Zhu-Tan City Cluster, Poyang Lake City Cluster, Cheng-Yu Urban Agglomeration, Guizhou City Cluster and Yunnan City Cluster. The average values and rankings of eco-efficiency urban agglomerations of the Yangtze River Economic Belt in four representative years, 2005, 2010, 2015 and 2019, are listed in Table 3.

As we can see from Table 3, the overall eco-efficiency of the Yangtze River Economic Belt shows an obvious upward trend, and the top three are entitled to the Yangtze River Delta Urban Agglomeration, Wuhan City Circle and Chang-Zhu-Tan City Cluster in recent years. The problem of water eutrophication in Poyang Lake and other watersheds is growing seriously, as the number of haze days in the central cities within Poyang Lake city cluster drastically. Although the ecological efficiency of the Poyang Lake city cluster has a rising trend in recent years, it is concerning that the rise is small, with a dropped ranking due to the unimproved extensive development mode. Cheng-Yu Urban Agglomeration has enjoyed the region with the highest industrialization and urbanization level in the upper reaches of the Yangtze River. However, the extensive development mode of industry has aggravated the structural pollution in the watershed, leading to a low ecological efficiency. With more support from national policies in recent years, especially the Development Plan of Cheng-Yu Urban Agglomeration issued by the State in 2016, it has resulted in the basic formation of an ecological security pattern and environmental zoning governance system, as well as the efficient utilization of resources with the ecological efficiency greatly improved in Cheng-Yu Urban Agglomeration, which ranked fourth in 2019. Even though the Guizhou City Cluster and Yunnan City Cluster were assigned to 19 urban clusters of key cultivation in China with a big improvement in eco-efficiency during these years, it was not high enough, as the average values of the eco-efficiency and rank were relatively behind compared to several city clusters of the Yangtze River Economic Belt, limited by the shortage of geographical location and natural endowment.

#### 4.1.2. Spatial Dynamic Analysis

**Figure 2:**
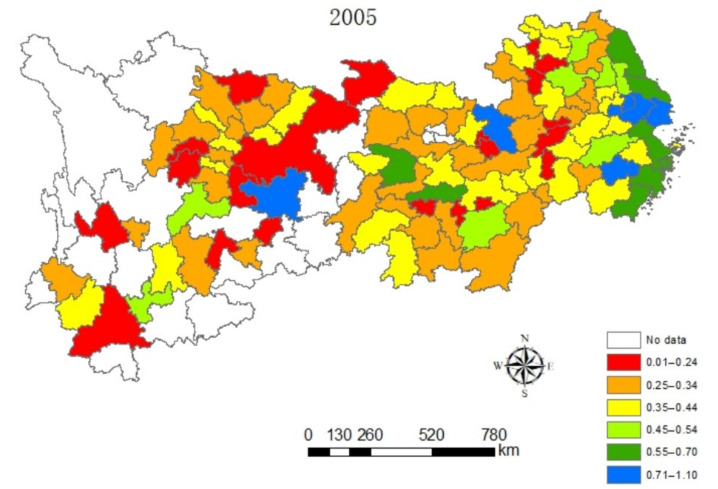
Spatial distribution characteristics of the eco-efficiency in urban clusters of the Yangtze River Economic Zone in 2005.

**Figure 3:**
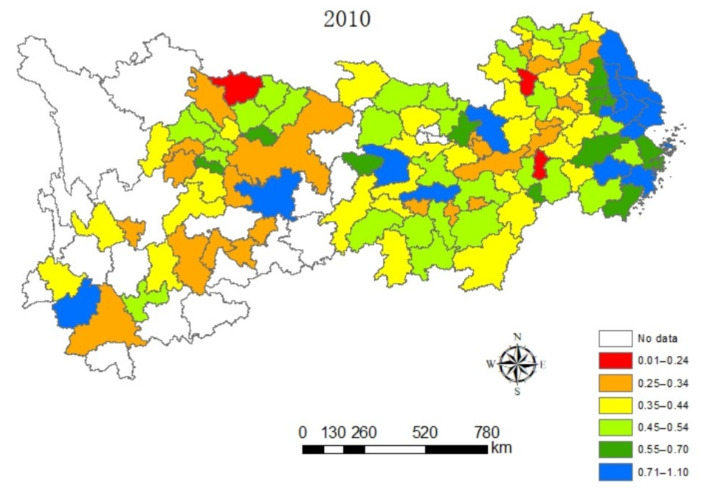
Spatial distribution characteristics of the eco-efficiency in urban clusters of the Yangtze River Economic Zone in 2010.

**Figure 4:**
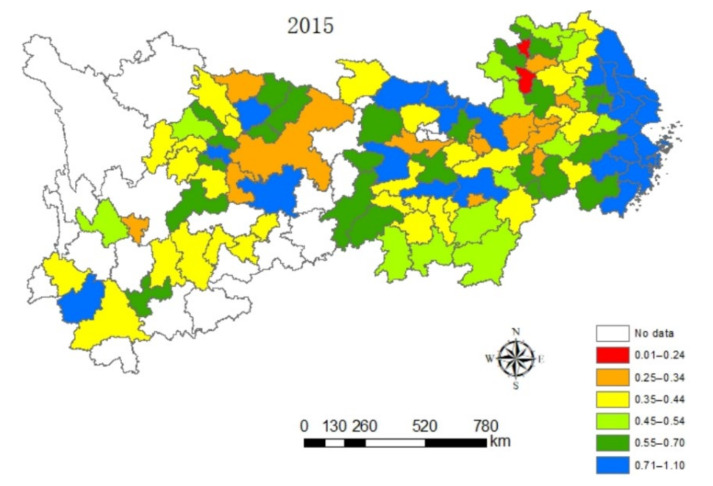
Spatial distribution characteristics of the eco-efficiency in urban clusters of the Yangtze River Economic Zone in 2015.

**Figure 5:**
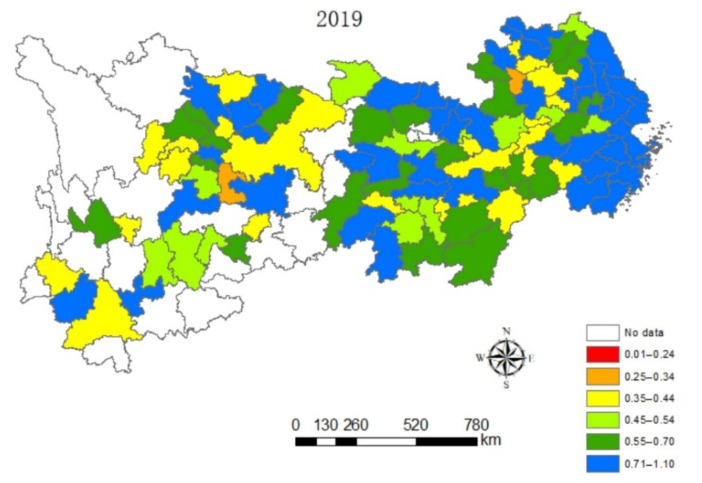
Spatial distribution characteristics of the eco-efficiency in urban clusters of the Yangtze River Economic Zone in 2019.

In this paper, the value of eco-efficiency of cities along the Yangtze River Economic Belt are naturally graded in a five-year phase by ArcGIS software, as can be seen from Figure 2, Figure 3, Figure 4 and Figure 5, which contain the visualization of the natural gradation of eco-efficiency of each city in 2005, 2010, 2015 and 2019. Influenced by technological progress and support from green policies of the government, the extensive energy utilization mode and economic growth mode of the Yangtze River Economic Belt become better and better. It can be seen that the eco-efficiency of urban clusters of the Yangtze River Economic Belt gradually improved on the whole from 2005 to 2019, but the differences in eco-efficiency among the regions are very significant, and sudden changes happen at the four time points. For one, located in the lower reaches of the Yangtze River, Shanghai as the central city, accompanied by Hangzhou, Nanjing and Hefei as the three sub-central cities, and the Yangtze River Delta Urban Agglomeration is one of the most economically developed regions in China at present, which has one of the largest energy-saving and environmental protection industry clusters in China. As usual, it leads the nation on an industrial basis, a development level, research strength and innovation level. The energy-saving and environmental protection cluster in Yangtze River Delta Urban Agglomeration are believed to be a way of relieving the ecological and environmental pressure in the region and promoted industrial transformation, with the realization of the coordination between economic development and environmental protection from 2005 to 2019. Next, the phenomenon of the overexploitation of resources in the urban cluster around Poyang Lake has brought a high cost for the resources and environment for regional economic development, coupled with a low level of environmental protection and an extensive economic development mode. Thirdly, the Wuhan City Circle and Chang-Zhu-Tan City Cluster situated in the middle reaches of the Yangtze River are supposed to be important supply bases of China’s manufacturing and energy, with a heavier task of energy-saving and emission reduction. Meanwhile, the Wuhan City Circle and Chang-Zhu-Tan City Cluster play a role as transferring land loaded with high energy-consuming and high-polluting industries in the Yangtze River Delta, burdened with heavier environmental protection. In 2005, the ranks of sixth and fourth in ecological efficiency were entitled to the Wuhan City Circle and Chang-Zhu-Tan City Cluster, respectively. In December 2007, the National Development and Reform Commission approved the Wuhan City Circle and the Chang-Zhu-Tan City Cluster as national resource-saving and environment-friendly society construction comprehensive supporting reform pilot zones. Benefiting from national policies of the pilot zones, more efforts have been made to form institutions and mechanisms that are conducive to energy and resource conservation and ecological environmental protection. The efforts of green innovation are done more to produce green and differentiated products for local enterprises and stimulate new market demands to achieve a win–win situation between economic efficiency and environmental protection. As a result, the rank of eco-efficiency in the Wuhan City Circle and Chang-Zhu-Tan City Cluster have increased significantly from 2005 to 2019 only after the Yangtze River Delta. Fourth, as the upper reaches of the Yangtze River, the Cheng-Yu Urban Agglomeration, Guizhou City Cluster and Yunnan City Cluster are the key areas of Western development policy in China. As a result of the support from various policies, such as increasing the capital investment, perfecting the investment environment, attracting talents and develop science and technology and education under relatively small influence of the industrial economy, it is expected to have a gradual improvement of the ecological efficiency from 2005 to 2019, whereas there is still more room for improving the eco-efficiency of these regions when released from the geographical location, the constraints of natural endowment, the relative lack of human resources, weak innovation capacity and low environmental governance.

### 4.2. Empirical Results and Analysis

The econometric Model (7), Model (8) and Model (9) are estimated by the two-step systematic generalized moment estimation method (two-step SYS-GMM), respectively. A reasonable SYS-GMM regression model is required to pass two tests: one is the second-order serial correlation test for random perturbation of the difference equation proposed by Arellano and Bond, and the other is the Sargan test for the over-identification of instruments. The estimate results are reported in Table 4 and Table 5. It can be seen that, for all models, there is first-order autocorrelation in the differences of the disturbance terms without second-order autocorrelation, indicating that the original hypothesis of no autocorrelation in the disturbance terms is accepted. In addition, the corresponding *p*-values by the Sargan test are all greater than 0.05, implying that the instruments are valid. The coefficient for ${\mathrm{EE}}_{t-1}$ was positive and significant at the 1% level, which means that the increase of each unit in ${\mathrm{EE}}_{t-1}$ induced an increase in the current EE. The next is to analyze the practical significance of the estimation results based on the coefficients for core explanatory variables and control variables.

#### 4.2.1. Green Technological Innovation Term

As shown in Table 5 column1, the coefficient for ln (tgreen + 1) was positive and significant at the 5% level in the downstream region, while it was not significant in the overall Yangtze River Economic Belt, midstream region and downstream region. The principal reason is that more investment costs of green technological innovations are too risky to implement at the initial stage. Only a few green industry enterprises with large-scale and strong capital are allowed to try higher prices for green products, corresponding to scenario 1 in the second part of this paper. As for the downstream region, the number of green industry enterprises capable of trying green innovation is sufficient enough to drive the overall green technological innovation to a certain level, which is a beginning to promote the local ecological efficiency in accordance with scenario 2 in the second part, which verifies Hypothesis 1.

#### 4.2.2. Interactive Term of Green Technological Innovation and Environmental Regulation

As shown in Table 4, column 2, the coefficients for ln (tgreen + 1) × reg is positive and significant at the 5% and 10% levels, respectively in the overall Yangtze River Economic Belt and midstream region, indicating that the enterprises’ willingness to innovate green technology is much more motivated by formal environmental regulation and the positive regulation effect between green technological innovation and ecological efficiency, in accordance with scenario 1 in the second part of this paper. The coefficients for ln (tgreen + 1) × reg were not significant in the upstream and downstream regions, which illustrates that formal environmental regulation is not appropriate for the development of local enterprises in the upstream and downstream regions, and the incentive effect on the local enterprises’ green technological innovations was not significant. Therefore, it failed to positively regulate the relationship between green technological innovation and green development, in accordance with the second case of formal environmental regulation in scenarios 1 and 2, respectively, in the second part of the paper. As for column 3, the coefficients for ln (tgreen + 1) × ireg are all positive and significant at the 1%, 1%, 10% and 5% levels, respectively, in the four regions, implying that informal environmental regulation can positively regulate the relationship between green technological innovation and green development in the four regions, which verifies Hypothesis 2.

Combining the coefficients for ln (tgreen + 1), ln (tgreen + 1) × reg and ln (tgreen + 1) × ireg, further results can be obtained: green technological innovation in the downstream region reached a certain level to significantly make an improvement of the local eco-efficiency. The purpose of the formal environmental regulation policy taken in the overall Yangtze River Economic Belt and the middle regions was to ultimately encourage local enterprises to engage in green technological innovation and guide local enterprises to find the right way for clean energy, clean production technologies and end-of-pipe treatment technology innovation and, more importantly, to regulate green technological innovation and green development, except for the upstream and downstream regions due to the unsuitable intensity of local formal environmental regulations for the development of local economies and enterprises. As for informal environmental regulation, it is certainly true that the awareness of environmental protection can help to form a behavior featuring green consumption, which is related to sending signals to enterprises for expanding the output of green products to realize green economic development in the overall Yangtze River Economic Belt upstream, midstream and downstream. Consequently, positive regulation makes sense to the relationship between green technological innovation and green development after being exposed to informal environmental regulation. 

#### 4.2.3. Control Variables

As shown in Table 4 and Table 5, the sign of coefficients for the control variables are basically the same in the three models; it is natural to take the estimation results in Model (6) as an example. Firstly, the coefficients for gov are negative and significant at the 1% level in the overall Yangtze River Economic Belt and downstream region, which means that government intervention has a negative effective on the local green development. Besides, the coefficients for gov were not significant in the upstream and midstream regions, with a meaning of no significant correlation with green development in the upstream and midstream regions. Second, the fact that the coefficients for open are all not significant in the four regions illustrates that green development is not sensitive to open. Third, the coefficients for r_gdp are all negative and significant in the four regions, indicating that the regional green development is stunted by the way of extensive economic growth mode. Fourth, the coefficients for structure are both negative and significant at the 5% and 10% levels, respectively, in the overall Yangtze River Economic Belt and the downstream regions, showing a decrease of the green development level with structure in the overall Yangtze River Economic Belt and the downstream regions, while the coefficients for structure are not significant in the upstream and midstream regions, indicating that green development is not sensitive to structure in the upstream and midstream regions. Fifth, the coefficients for lnpgdp are all positive and significant in the four regions, so that the green development level increases with lnpgdp. Sixth, the coefficients for r_urb are all extremely small and not significant in the four regions, representing that green development is not sensitive to r_urb.

## 5. Robustness Test

### 5.1. Changing the Measurement Method of Core Explanatory Variable

Another method to measure green technological innovation is represented as the natural logarithm of (1 + the number of green patents granted by prefecture-level cities in the current year), with the results of Model (7), Model (8) and Model (9) shown in Table 6 and Table 7, finding that the symbols and significance of the core independent variables remain unchanged, which gives excellent evidence to prove the main conclusions.

### 5.2. Changing the Measurement Method of Core Dependent Variable

In order to further verify the reliability of the results in this paper, another way to measure green development is shown in a robustness test by replacing green development with energy consumption per unit of GDP with reference to Wen et al. [25]. The specific formula is as follows.

(10) $$\mathrm{Energy}\mathrm{Cpu}\mathrm{OPV}=\frac{\sum_{k=1}^{N}\mathrm{kEnergy}C\times \mathrm{CtHCC}}{\mathrm{GDP}}$$

where Energy Cpu OPV represents “Energy consumption per unit output value” and “the kth energy consumption” for kEnergy C and “Corresponding to the heat conversion coefficient” for CtHCC. The composite of the energy index includes three main energy sources: electricity, gas and LPG. The results of the test are shown in Table 8 and Table 9. 

In replacement of the dependent variable metric and no change in the symbols and significance of the core independent variables, the main conclusions of this paper are proven to be correct.

### 5.3. Exclusion of Singular Value Effects

Due to the large number of independent variables involved in this paper and the large sample size with macro data, some biases may appear in both the process of collection and the measurements. For the purpose of alleviating the unstable results caused by this bias, all of the continuous variables were subjected to tailing below the 1% quantile and above the 99% quantile. After the tailing process, the regression results are shown in Table 10 and Table 11, and it can be seen that nothing has changed for the symbols and significances of the core independent variables, which stand for the main conclusions of this paper.

In summary, the experimental conclusions are given to show the good robustness in this paper.

## 6. Conclusions and Recommendations

Green technological innovation is the cornerstone of regional green development in China, and environmental regulation is likely to be the tipping point to guide regional green technological innovation. The purpose of this paper was to examine whether the current green technological innovation can effectively support local green development or if local formal and informal environmental regulations can positively regulate the relationship between green technological innovation and green development in each city cluster of the Yangtze River Economic Zone. As an integration of resource conservation, environmental protection and economic output, the index of eco-efficiency was identified to be a measurement of regional green development by the super-efficiency SBM window model to evaluate the eco-efficiency of 108 cities at the prefecture level and above of the Yangtze River Economic Belt. The systematic GMM model was used to figure out the relationship between environmental regulation, regional eco-efficiency and green technological innovation with a series of robustness tests. The conclusions are drawn below.

First, there was a growing tendency among the overall green development level of the Yangtze River Economic Belt from 2005 to 2019, accompanied by obvious regional differences. As an economically developed region, the Yangtze River Delta Urban Agglomeration stays ahead in the field of the green development. Even though the Wuhan City Circle and Chang-Zhu-Tan City Cluster are dominated by heavy industry with an extensive economic growth mode, the regional green development has been greatly improved owing to the support of the national policy of comprehensive reform pilot zone for the construction of two types of societies. Recently, the economic development mode has made certain improvements to the development of the Poyang Lake city Cluster, Cheng-Yu Urban Agglomeration, Guizhou City Cluster and Yunnan City Cluster. However, the level of green development has yet to be strengthened.

Second, no obvious improvement of green technological innovation was performed on green development in the overall the Yangtze River Economic Zone and the upstream and midstream levels, but the downstream areas had a significant positive improvement.

Third, formal environmental regulations positively regulate the relationship between green technological innovation and green development in the overall Yangtze River Economic Belt and the midstream region, but have no significant effect in the upstream and downstream regions, while informal environmental regulations showed a significant positive regulation effect in the overall Yangtze River Economic Belt and the upstream and midstream and downstream regions.

Based on the above findings, some helpful policy recommendations are put forward as follows:

Firstly, according to the results of the measurement of green development in various regions of the Yangtze River Economic Belt in this paper, it is urgent to establish a green development evaluation system learning from relevant international experience actively. Moreover, an integrated system is required to create, including tracking, a statistical monitoring and evaluation mechanism for green development evaluation and provide effective supports for international comparisons, formulating relevant policies in energy utilization, land and water conservation and utilization; pollution control; modes in production and lifestyle; the promotion of green development; etc. In addition, in view of the differences of the green development levels between cities in the Yangtze River Economic Belt being quite different, it is necessary to strengthen the cooperation in the allocation of resources and environmental elements and the construction of ecological civilization among cities to promote the coordinated development of urban agglomerations.

Secondly, according to the research conclusions on the relationship between green technological innovation and green development in this paper, for the upstream and midstream regions of the Yangtze River Economic Belt green technological innovation research and development on a large scale, more efforts should be paid to the training of talents, increase of investment in education and scientific research and policy-making to guide scientific researchers to devote themselves to the research and development of green technological innovation. However, for the downstream regions of the Yangtze River Economic Belt, emphasis is given to the supervision and guidance of the technological market to promote the transformation of green technological achievements, bringing the positive promotion role of green technological innovation into full play.

Thirdly, according to the research conclusions on the different adjustment effects of environmental regulations in the upstream, midstream and downstream regions of the Yangtze River Economic Belt, it is imperative to implement differentiated environmental regulation policies. In the upstream and downstream regions of the Yangtze River Economic Belt, it is effective to moderately increase the intensity of subsidy policies such as fiscal expenditures for environmental protection, while the work of further optimizing the use of various formal environmental regulation tools is needed and sensible in the mid region of that, together with strengthening the public environmental protection publicity and improving the environmental governance system to encourage them to actively participate in environmental protection. Moreover, the guidance of the public’s consumption of green products is conducive to give full play to the positive adjustment role of informal environmental regulations on the relationship between green technological innovation and green development, which is consistent with the recommendation by Zhou et al. [54], who confirmed that differentiated environmental governance policies must be implemented based on the heterogeneity of the economic development and technological characteristics of different provinces in China, and the recommendation by Feng and Chen [10], who suggested that the government should fully consider the adaptability of different types of environmental regulation in different regions and implement the policies and tools of environmental regulation.

Fourthly, it can be found that formal environmental regulation and informal environmental regulation go a long way to stimulate enterprises’ willingness for green technological innovation and positively adjust the relationship between green technological innovation and green development under certain conditions. Therefore, it is urgent and important for the government to build an environmental governance system in which the government for the leaders and enterprises for the main body get social organizations and the public together to shape the synergy of the whole society embracing the environment. The mobile Internet should be applied to mobilize the enthusiasm of all sectors of society and broaden the channels for the public to appeal for environmental protection, such as the environmental protection WeChat complaint platform. In short, it makes a difference to cultivate the enthusiasm of the public to participate in environmental management. Therefore, it is necessary to cultivate the public’s enthusiasm for participating in environmental management to work hard to form a “government–enterprise-public” tripartite governance situation and create a “community of environmental interest” that is coordinated, complementary, incentive and compatible.

### Limitations and Future Research

Although this study provides valuable insights, it has its limitations for further research. Firstly, due to the change in statistical caliber and the lack of data in some area, it was difficult to acquire parts of the evaluation index data, such as the Volume of Industrial Dust Emission. The same data item collected in different regions or years may lead to the problem of inconsistent scales or targets. Taking the caliber of exhaust gas emission as an example, sulfur dioxide was included in the first 3 years but not in the latter 3 years. Governments and scholars need to work together to standardize the statistical caliber of data to improve and revise eco-efficiency measurements in the future. Secondly, in our study, environmental regulation was divided into formal environmental regulation and informal environmental regulation, which are measured by a comprehensive index in empirical research. In further research, a further expansion of the types of environmental regulation is waiting to be discovered. According to the development of environmental regulation policies, such as administrative environmental regulation, market-based environmental regulation and public participation environmental regulation, in reality, more guiding conclusions can be obtained. Thirdly, in this paper, we took 108 prefecture-level and above cities of the Yangtze River Economic Belt as the research object, with some meaningful results. However, the situations in different regions of China may be different stories, such as the Beijing–Tianjin–Hebei region known as China’s “Capital Economic Circle” and the Pearl River Delta Economic Zone, named as one of the most dynamic economic zones in the Asia-Pacific region. In order to keep the consistency of the results, more attempts were done to expand further research by taking panel data from the Beijing–Tianjin–Hebei region and Pearl River Delta Economic Zone as the research objects.

## Figures and Tables

**Table 1:** The window analysis of a DMU in period T (d=3).

Windows	t=1	t=2	t=3	t=4	t=5	…	t=T−4	t=T−3	t=T−2	t=T−1	t=T
1	$E_{11}$	$E_{12}$	$E_{13}$								
2		$E_{21}$	$E_{22}$	$E_{23}$							
3			$E_{31}$	$E_{32}$	$E_{33}$						
…						…					
T−d−1							$E_{T-d-1,1}$	$E_{T-d-1,2}$	$E_{T-d-1,3}$		
T−d								$E_{T-d,1}$	$E_{T-d,2}$	$E_{T-d,3}$	
T−d+1									$E_{T-d+1,1}$	$E_{T-d+1,2}$	$E_{T-d+1,3}$
Average value											

**Table 2:** Eco-efficiency evaluation index system.

Tier 1 Indicators	Secondary Indicators	Tertiary Indicators (Unit)
Input	Capital investment	Investment in Fixed Assets (10,000 yuan)
	Resource input	Energy Consumption (10,000 tce)
		Water Supply (10,000 tons)
		Area of Land Used for Urban Construction (square kilometer)
		Employment (10,000 people)
Desirable output	Regional development indicators	Regional Gross Domestic Product (10,000 yuan)
Undesirable output	Environmental benefit indicators	Volume of Industrial Wastewater Discharged (10,000 tons)
		Volume of Sulphur Dioxide Emission (ton)
		Volume of Industrial Dust Emission (ton)

**Table 3:** Average value and ranking of the eco-efficiency of urban agglomerations of the Yangtze River Economic Belt.

Urban Agglomerations	2005	2010	2015	2019
Yangtze River Delta Urban Agglomeration	0.4855 (1)	0.6037 (1)	0.6607 (1)	0.822 (1)
Wuhan City Circle	0.3259 (6)	0.5369 (3)	0.6136 (2)	0.751 (2)
Chang-Zhu-Tan City Cluster	0.3734 (4)	0.5776 (2)	0.5788 (3)	0.748 (3)
Poyang Lake City Cluster	0.4416 (3)	0.4071 (7)	0.5014 (6)	0.586 (6)
Cheng-Yu Urban Agglomeration	0.3050 (7)	0.4120 (6)	0.4993 (7)	0.693 (4)
Guizhou City Cluster	0.3375 (5)	0.4694 (5)	0.5353 (5)	0.571 (7)
Yunnan City Cluster	0.4826 (2)	0.5023 (4)	0.5705 (4)	0.669 (5)

**Table 4:** Two-step SYS-GMM for Models (7), (8) and (9).

	Overall			Upstream		
Explanatory Variables	Model (7)	Model (8)	Model (9)	Model (7)	Model (8)	Model (9)
L.EE	1.713 *** (0.062)	1.708 *** (0.060)	1.709 *** (0.059)	1.794 *** (0.174)	1.796 *** (0.170)	1.780 *** (0.153)
ln (tgreen + 1)	0.007 (0.007)			0.018 (0.016)		
ln (tgreen + 1) × reg		0.017 ** (0.007)			0.021 (0.021)	
ln (tgreen + 1) × ireg			0.187 *** (0.043)			0.508 *** (0.119)
gov	−0.539 *** (0.116)	−0.515 *** (0.122)	−0.379 ** (0.112)	−0.036 (0.457)	−0.089 (0.372)	−0.290 (0.286)
open	0.372 (0.536)	0.137 (0.532)	0.168 (0.474)	3.543 (4.503)	2.541 (4.290)	0.874 (3.312)
r_gdp	−2.016 *** (0.300)	−1.791 *** (0.335)	−1.515 *** (0.300)	−1.740 ** (0.719)	−1.804 ** (0.754)	0.738 (0.545)
structure	−0.289 ** (0.137)	−0.288 ** (0.139)	0.222 (0.140)	−0.281 (0.333)	−0.220 (0.336)	−0.005 (0.219)
lnpgdp	0.117 *** (0.010)	0.120 *** (0.010)	0.131 *** (0.010)	0.142 *** (0.024)	0.140 *** (0.024)	0.167 *** (0.019)
r_urb	0.000 (0.000)	0.000 (0.000)	0.000 (0.000)	0.000 (0.000)	0.000 (0.000)	0.000 (0.000)
Wald chi2	714,781.6 (0.000)	704,860.5 (0.000)	2,084,070 (0.000)	3,156,767 (0.000)	4,011,887 (0.000)	7,188,350 (0.000)
AR (1)	−3.778 (0.000)	−3.746 (0.000)	−3.741 (0.000)	−2.367 (0.018)	−2.349 (0.019)	−2.326 (0.020)
AR (2)	0.974 (0.330)	0.918 (0.359)	0.814 (0.416)	1.478 (0.139)	1.502 (0.133)	1.355 (0.175)
Sargan test	98.517 (0.815)	97.706 (0.830)	98.872 (0.808)	26.595 (1.000)	26.233 (1.000)	27.915 (1.000)

Note: ***, ** and * indicate significance at the significance level of 1%, 5% and 10%, respectively. The parentheses in AR (1), AR (2) and the Sargan test are probability values for Prob > z, and the other parentheses refer to the standard errors of the clustering robustness.

**Table 5:** Two-step SYS-GMM for Models (7), (8) and (9).

	Midstream			Downstream		
Explanatory Variables	Model (7)	Model (8)	Model (9)	Model (7)	Model (8)	Model (9)
L.EE	1.657 *** (0.142)	1.630 *** (0.113)	1.606 *** (0.105)	1.710 *** (0.099)	1.732 *** (0.109)	1.732 *** (0.118)
ln (tgreen + 1)	0.003 (0.013)			0.014 ** (0.007)		
ln (tgreen + 1) × reg		0.031 * (0.015)			−0.004 (0.011)	
ln (tgreen + 1) × ireg			0.219 * (0.125)			0.092 ** (0.037)
gov	0.690 (0.888)	0.027 (0.337)	0.133 (0.435)	−0.801 *** (0.267)	−0.815 *** (0.273)	−0.808 *** (0.233)
open	−3.274 (4.582)	−1.122 (2.066)	−0.842 (2.634)	−0.120 (0.652)	0.031 (0.422)	−0.144 (0.482)
r_gdp	−1.461 ** (0.641)	−1.589 *** (0.602)	−1.609 ** (0.648)	−1.026 ** (0.419)	−1.333 *** (0.449)	−0.677 (0.434)
structure	−0.380 (0.339)	−0.166 (0.302)	−0.217 (0.251)	−0.457 * (0.249)	−0.458 ** (0.233)	−0.391 (0.254)
lnpgdp	0.139 *** (0.042)	0.145 *** (0.020)	0.137 *** (0.23)	0.125 *** (0.021)	0.19 *** (0.020)	0.133 *** (0.019)
r_urb	0.255 (0.545)	0.217 (0.328)	0.083 (0.414)	0.297 (0.239)	0.244 (0.238)	0.249 (0.230)
Wald chi2	1951.693 (0.000)	3248.390 (0.000)	3499.768 (0.000)	3539.605 (0.000)	3430.743 (0.000)	3688.362 (0.000)
AR (1)	−3.096 (0.002)	−2.884 (0.004)	−3.003 (0.003)	−2.017 (0.043)	−2.019 (0.042)	−2.024 (0.041)
AR (2)	1.439 (0.150)	1.323 (0.186)	1.426 (0.154)	1.485 (0.138)	1.364 (0.173)	1.470 (0.142)
Sargan test	24.968 (1.000)	95.322 (0.358)	28.734 (0.984)	31.323 (0.962)	32.812 (0.942)	30.955 (0.966)

Note: ***, ** and * indicate significance at the significance level of 1%, 5% and 10%, respectively. The parentheses in AR (1), AR (2) and the Sargan test are probability values for Prob > z, and the other parentheses refer to the standard errors of the clustering robustness.

**Table 6:** Robustness tests: estimation results for replacing the core explanatory variables.

	Overall			Upstream		
Explanatory Variables	Model (7)	Model (8)	Model (9)	Model (7)	Model (8)	Model (9)
L.EE	1.744 *** (0.072)	1.761 *** (0.070)	1.725 *** (0.063)	1.789 *** (0.129)	1.829 *** (0.197)	1.760 *** (0.091)
ln (tgreen + 1)	0.001 (0.005)			0.013 (0.010)		
ln (tgreen + 1) × reg		0.012 ^*^ (0.007)			0.018 (0.016)	
ln (tgreen + 1) × ireg			0.161 *** (0.035)			0.124 *** (0.035)
gov	−0.516 *** (0.108)	−0.547 *** (0.147)	−0.417 *** (0.105)	−0.177 (0.241)	−0.025 (0.304)	−0.019 (0.105)
open	0.525 (0.529)	0.306 (0.455)	0.368 (0.529)	2.963 (3.550)	1.619 (7.287)	1.327 (2.529)
r_gdp	−2.225 *** (0.310)	−1.815 *** (0.303)	−1.721 *** (0.288)	−2.211 *** (0.517)	−1.798 ** (0.748)	−1.631 *** (0.450)
structure	−0.216 (0.144)	−0.226 (0.159)	−0.171 (0.135)	−0.040 (0.219)	−0.189 (0.284)	−0.164 (0.223)
lnpgdp	−0.118 *** (0.010)	−0.123 *** (0.011)	−0.129 *** (0.009)	−0.142 *** (0.018)	−0.141 *** (0.024)	−0.140 ***(0.023)
r_urb	0.000 *** (0.000)	0.000 *** (0.000)	0.000 *** (0.000)	0.000 *** (0.000)	0.000 *** (0.000)	0.000 *** (0.000)
Wald chi2	584,980.1 (0.000)	673,878.9 (0.000)	1,083,022 (0.000)	4,887,047 (0.000)	2,405,002 (0.000)	8,936,472 (0.000)
AR (1)	−3.931 (0.000)	−3.133 (0.002)	−3.078 (0.002)	−2.576 (0.010)	−2.365 (0.018)	−2.193 (0.028)
AR (2)	0.945 (0.345)	0.815 (0.415)	0.715 (0.475)	1.487 (0.137)	1.438 (0.150)	1.001 (0.317)
Sargan test	99.936 (0.786)	99.935 (0.786)	99.667 (0.791)	25.194 (0.996)	23.343 (1.000)	27.454 (1.000)

Note: ***, ** and * indicate significance at the significance level of 1%, 5% and 10%, respectively. The parentheses in AR (1), AR (2) and the Sargan test are probability values for Prob > z, and the other parentheses refer to the standard errors of the clustering robustness.

**Table 7:** Robustness test: estimation results for replacing the core explanatory variables.

	Midstream			Downstream		
Explanatory Variables	Model (7)	Model (8)	Model (9)	Model (7)	Model (8)	Model (9)
L.EE	1.630 *** (0.089)	1.629 ***(0.098)	1.611 ***(0.102)	1.722 *** (0.116)	1.725 ***(0.122)	1.693 *** (0.106)
ln (tgreen + 1)	0.004 (0.011)			0.004 ** (0.002)		
ln (tgreen + 1) × reg		0.019 * (0.009)			−0.007 (0.008)	
ln (tgreen + 1) × ireg			0.158 * (0.081)			0.042 ** (0.020)
Gov	0.282 (0.392)	0.606 (0.564)	0.112 (0.443)	−0.691 *** (0.251)	−0.613 ** (0.251)	−0.690 ** (0.277)
open	−0.242 (1.812)	0.196 (2.235	−0.974 (2.308)	−0.308 *** (0.507)	−0.387 (0.648)	−0.553 (0.672)
r_gdp	−2.035 *** (0.715)	−1.671 *** (0.646)	−1.955 ** (0.566)	−0.839 * (0.478)	−0.962 ** (0.449)	−0.687 (0.485)
structure	−0.234 (0.286)	−0.118 (0.260)	−0.142 (0.251)	−0.481 (0.295)	−0.415 (0.293)	−0.512 * (0.279)
lnpgdp	−0.140 *** (0.027)	−0.160 *** (0.029)	−0.135 *** (0.018)	0.131 *** (0.023)	−0.134 *** (0.022)	−0.129 *** (0.022)
r_urb	0.276 (0.378)	0.296 (0.379)	0.119 (0.440)	0.391 * (0.229)	0.404 * (0.243)	0.358 (0.282)
Wald chi2	2958.146 (0.000)	2555.434 (0.000)	2678.346 (0.000)	3559.347 (0.000)	3989.746 (0.000)	2892.464 (0.000)
AR (1)	−2.944 (0.003)	−3.101 (0.002)	−3.324 (0.001)	−1.973 (0.048)	−2.187 (0.029)	−2.069 (0.039)
AR (2)	1.572 (0.116)	1.536 (0.124)	1.601 (0.109)	1.447 (0.148)	1.533 (0.125)	1.521 (0.128)
Sargan test	30.463 (0.971)	28.110 (0.987)	31.365 (0.961)	31.816 (0.956)	30.951 (0.966)	31.539 (0.959)

Note: ***, ** and * indicate significance at the significance level of 1%, 5% and 10%, respectively. The parentheses in AR (1), AR (2) and the Sargan test are probability values for Prob > z, and the other parentheses refer to the standard errors of the clustering robustness.

**Table 8:** Robustness tests: estimation results for replacing the dependent variable.

	Overall			Upstream		
Explanatory Variables	Model (7)	Model (8)	Model (9)	Model (7)	Model (8)	Model (9)
L.EE	3.991 *** (1.079)	4.083 *** (1.139)	4.010 *** (1.227)	2.502 ** (1.253)	2.630 ** (1.308)	2.600 ** (1.298)
ln (tgreen + 1)	0.037 * (0.022)			−0.060 (0.038)		
ln (tgreen + 1) × reg		0.112 *** (0.028)			−0.007 (0.058)	
ln (tgreen + 1) × ireg			0.741 *** (0.162)			0.187 *** (0.062)
gov	−1.961 *** (0.631)	−1.823 *** (0.671)	−1.309 ** (0.664)	−2.894 (2.086)	−2.711 (1.668)	−2.961 (2.376)
open	3.202 * (1.715)	3.221 * (1.776)	3.293 * (1.694)	−8.251 (8.850)	−7.351 (8.106)	−7.998 (9.431)
r_gdp	−6.558 ** (1.206)	−5.449 *** (1.151)	−5.034 *** (1.096)	−4.775 (3.013)	−3.977 (2.878)	−3.352 (2.932)
structure	0.747 (0.641)	0.796 (0.647)	1.158 * (0.604)	0.907 (1.112)	1.223 (1.049)	1.107 (1.198)
lnpgdp	−0.56 *** (0.029)	−0.285 *** (0.011)	−0.319 *** (0.029)	−0.440 *** (0.129)	−0.478 *** (0.117)	−0.467 *** (0.123)
r_urb	0.000 *** (0.000)	0.000 *** (0.000)	0.000 *** (0.000)	4.217 ** (1.794)	4.032 ** (1.762)	3.768 ** (1.769)
Wald chi2	296,887.9 (0.000)	318,523.7 (0.000)	799,877 (0.000)	1,884,554 (0.000)	2,458,586 (0.000)	2,422,324 (0.000)
AR (1)	−2.111 (0.035)	−2.275 (0.023)	−2.088 (0.037)	−2.655 (0.008)	−2.617 (0.009)	−2.623 (0.009)
AR (2)	0.855 (0.392)	0.621 (0.534)	0.492 (0.623)	−0.145 (0.885)	0.144 (0.885)	−0.151 (0.880)
Sargan test	93.360 (0.899)	97.729 (0.830)	98.973 (0.806)	26.322 (1.000)	26.210 (1.000)	25.556 (1.000)

Note: ***, ** and * indicate significance at the significance level of 1%, 5% and 10%, respectively. The parentheses in AR (1), AR (2) and the Sargan test are probability values for Prob > z, and the other parentheses refer to the standard errors of the clustering robustness.

**Table 9:** Robustness test: estimation results of replacing the dependent variable.

	Midstream			Downstream		
Explanatory Variables	Model (7)	Model (8)	Model (9)	Model (7)	Model (8)	Model (9)
L.EE	4.411 *** (0.733)	4.728 *** (1.103)	4.318 *** (0.808)	11.456 *** (1.231)	11.114 *** (1.142)	11.139 ***(1.158)
ln (tgreen + 1)	0.079 ** (0.039)			0.047 ** (0.022)		
ln (tgreen + 1) × reg		0.163 ** (0.066)			0.056 ** (0.025)	
ln (tgreen + 1) × ireg			0.984 *** (0.371)			0.340 *** (0.111)
gov	−0.650 (1.239)	−0.123 (1.327)	0.079 (1.263)	−1.593 (1.198)	−1.502 (1.190)	−1.198 (1.164)
open	−0.008 (0.118)	0.511 (13.984)	−2.032 (1.366)	1.822 (2.776)	1.843 (2.815)	1.917 (2.384)
r_gdp	−2.571 (2.308)	−1.957 (2.127)	−1.406 (2.010)	−2.707 ** (1.253)	−2.585 ** (1.196)	−2.611 ** (1.236)
structure	−0.054 (0.811)	0.006 (0.780)	−0.353 (0.833)	−0.427 (0.606)	−0.484 (0.649)	−0.081 (0.675)
lnpgdp	−0.293 *** (0.047)	−0.321 *** 0.056)	−0.339 *** (0.050)	−0.386 *** (0.089)	−0.383 *** (0.086)	−0.401 *** (0.096)
r_urb	0.000 ** (0.000)	0.000 ** (0.000)	0.000 ** (0.000)	1.695 * (0.868)	1.710 ** (0.757)	1.575 ** (0.782)
Wald chi2	1704.243 (0.000)	1433.249 (0.000)	1436.658 (0.000)	6981.781 (0.000)	6067.001 (0.000)	7972.238 (0.000)
AR (1)	−2.143 (0.032)	−2.226 (0.026)	−2.065 (0.039)	−4.043 (0.000)	−4.160 (0.000)	−4.136 (0.050)
AR (2)	0.773 (0.440)	0.917 (0.359)	0.849 (0.396)	0.282 (0.778)	0.073 (0.942)	0.125 (0.900)
Sargan test	32.933 (1.000)	32.799 (1.000)	26.763 (1.000)	38.350 (1.000)	38.253 (1.000)	38.715 (1.000)

Note: ***, ** and * indicate significance at the significance level of 1%, 5% and 10%, respectively. The parentheses in AR (1), AR (2) and the Sargan test are probability values for Prob > z, and the other parentheses refer to the standard errors of the clustering robustness.

**Table 10:** Robustness test: estimation results excluding the effects of singular values.

	Overall			Upstream		
Explanatory Variables	Model (7)	Model (8)	Model (9)	Model (7)	Model (8)	Model (9)
L.EE	1.718 *** (0.063)	1.716 *** (0.064)	1.712 *** (0.057)	1.815 *** (0.180)	1.800 *** (0.180)	1.758 *** (0.150)
ln (tgreen + 1)	0.006 (0.007)			0.015 (0.016)		
ln (tgreen + 1) × reg		0.016 * *(0.008)			0.021 (0.021)	
ln (tgreen + 1) × ireg			0.200 *** (0.042)			0.518 *** (0.121)
gov	−0.532 *** (0.123)	−0.535 *** (0.124)	−0.342 *** (0.112)	−0.014 (0.529)	−0.109 (0.552)	−0.254 (0.489)
open	0.392 (0.548)	0.240 (0.569)	0.167 (0.478)	3.550 (4.041)	2.328 (4.079)	1.046 (4.417)
r_gdp	−2.112 *** (0.315)	−1.868 *** (0.340)	−1.493 *** (0.289)	−1.768 ** (0.700)	−1.719 ** (0.776)	−0.705 (0.546)
structure	−0.280 ** (0.142)	−0.283 ** (0.143)	−0.208 (0.134)	−0.311 (0.321)	−0.290 (0.330)	−0.019 (0.221)
lnpgdp	−0.116 *** (0.011)	−0.119 *** (0.010)	−0.133 *** (0.009)	−0.141 *** (0.024)	−0.138 *** (0.025)	−0.167 *** (0.023)
r_urb	0.000 *** (0.000)	0.000 *** (0.000)	0.000 *** (0.000)	0.000 (0.000)	0.000 (0.000)	0.000 (0.000)
Wald chi2	729,509.6 (0.000)	732,572.6 (0.000)	2,316,610 (0.000)	2,786,905 (0.000)	4,067,023 (0.000)	8,071,266 (0.000)
AR (1)	−3.794 (0.000)	−3.781 (0.000)	−3.740 (0.000)	−2.315 (0.021)	−2.298 (0.022)	−2.300 (0.021)
AR (2)	0.946 (0.344)	0.816 (0.414)	0.716 (0.474)	1.419 (0.156)	1.450 (0.147)	1.260 (0.208)
Sargan test	100.813 (0.767)	99.960 (0.785)	98.354 (0.818)	26.045 (1.000)	25.978 (1.000)	27.972 (1.000)

Note: ***, ** and * indicate significance at the significance level of 1%, 5% and 10%, respectively. The parentheses in AR (1), AR (2) and the Sargan test are probability values for Prob > z, and the other parentheses refer to the standard errors of the clustering robustness.

**Table 11:** Robustness test: estimation results excluding the effects of singular values.

	Midstream			Downstream		
Explanatory Variables	Model (7)	Model (8)	Model (9)	Model (7)	Model (8)	Model (9)
L.EE	1.673 *** (0.148)	1.631 *** (0.107)	1.649 ***(0.095)	1.716 *** (0.102)	1.734 *** (0.097)	1.727 *** (0.119)
ln (tgreen + 1)	0.002 (0.013)			0.014 ** (0.007)		
ln (tgreen + 1) × reg		0.031 * (0.016)			−0.005 (0.011)	
ln (tgreen + 1) × ireg			0.325 *** (0.102)			0.088 ** (0.038)
gov	0.641 (0.914)	0.027 (0.311)	0.255 (0.353)	−0.820 ***(0.273)	−0.863 *** (0.262)	−0.837 ***(0.232)
open	−3.204 (4.790)	−1.082 (1.804	−0.632 (2.035)	−0.173 (0.643)	−0.159 (0.480)	−0.206 (0.507)
r_gdp	−1.448 * (0.766)	−1.468 ** (0.673)	−1.169 ** (0.561)	−1.082 ** (0.422)	−1.317 *** (0.486)	−0.726 * (0.435)
structure	−0.283 (0.340)	−0.107 (0.282)	−0.070 (0.232)	−0.456 * (0.244)	−0.468 ** (0.236)	−0.392 (0.257)
lnpgdp	−0.145 *** (0.037)	−0.151 *** (0.019)	−0.156 *** (0.015)	−0.123 *** 0.020)	−0.116 *** (0.020)	−0.130 ***(0.020)
r_urb	0.283 (0.619)	0.244 (0.389)	0.080 (0.390)	0.277 (0.236)	0.220 (0.220)	0.227 (0.230)
Wald chi2	1821.494 (0.000)	3153.963 (0.000)	3732.763 (0.000)	3257.005 (0.000)	3718.475 (0.000)	3909.4 (0.000)
AR(1)	−3.006 (0.003)	−2.922 (0.003)	−2.872 (0.004)	−2.131 (0.033)	−1.964 (0.049)	−2.040 (0.041)
AR(2)	1.431 (0.152)	1.352 (0.176)	1.335 (0.182)	1.500 (0.134)	1.381 (0.167)	1.486 (0.137)
Sargan test	26.922 (1.000)	30.466 (0.971)	29.802 (0.976)	31.253 (0.963)	32.216 (0.951)	30.541 (0.970)

Note: ***, ** and * indicate significance at the significance level of 1%, 5% and 10%, respectively. The parentheses in AR (1), AR (2) and the Sargan test are probability values for Prob > z, and the other parentheses refer to the standard errors of the clustering robustness.

## Data Availability

Most of the data and programs in this paper can be published on the Internet.
